# Motor Adaptation Impairment in Chronic Cannabis Users Assessed by a Visuomotor Rotation Task

**DOI:** 10.3390/jcm8071049

**Published:** 2019-07-18

**Authors:** Ivan Herreros, Laia Miquel, Chrysanthi Blithikioti, Laura Nuño, Belen Rubio Ballester, Klaudia Grechuta, Antoni Gual, Mercè Balcells-Oliveró, Paul Verschure

**Affiliations:** 1 SPECS Lab, Universitat Pompeu Fabra, 08002 Barcelona, Spain; 2 GRAC, Grup de Recerca en Addiccions Clínic, Villarroel, 170 08036 Barcelona, Spain; 3 IDIBAPS, Institut d’Investigacions Biomèdiques August Pi i Sunyer, Villarroel, 170 08036 Barcelona, Spain; 4 IBEC, Institute for Biomedical Engineering of Catalonia, Universitat Politècnica de Catalunya, 08028 Barcelona, Spain; 5 ICREA, Institució Catalana de Recerca i Estudis Avançats, Passeig Lluís Companys, 08010 Barcelona, Spain

**Keywords:** cerebellum, cannabis, implicit motor learning, motor adaptation, visuomotor rotation

## Abstract

Background—The cerebellum has been recently suggested as an important player in the addiction brain circuit. Cannabis is one of the most used drugs worldwide, and its long-term effects on the central nervous system are not fully understood. No valid clinical evaluations of cannabis impact on the brain are available today. The cerebellum is expected to be one of the brain structures that are highly affected by prolonged exposure to cannabis, due to its high density in endocannabinoid receptors. We aim to use a motor adaptation paradigm to indirectly assess cerebellar function in chronic cannabis users (CCUs). Methods—We used a visuomotor rotation (VMR) task that probes a putatively-cerebellar implicit motor adaptation process together with the learning and execution of an explicit aiming rule. We conducted a case-control study, recruiting 18 CCUs and 18 age-matched healthy controls. Our main measure was the angular aiming error. Results—Our results show that CCUs have impaired implicit motor adaptation, as they showed a smaller rate of adaptation compared with healthy controls (drift rate: 19.3 +/− 6.8° vs. 27.4 +/− 11.6°; t(26) = −2.1, *p* = 0.048, Cohen’s *d* = −0.8, 95% CI = (−1.7, −0.15)). Conclusions—We suggest that a visuomotor rotation task might be the first step towards developing a useful tool for the detection of alterations in implicit learning among cannabis users.

## 1. Introduction

Cannabis is the most consumed illicit drug of abuse worldwide, ranking right after alcohol and tobacco [1,2]. The effects of the principal active component of cannabis, Delta9-tetrahydrocannabinol (THC), on the brain are still not well characterized. The cerebellum has several motor and cognitive functions [3] and has been suggested as a crucial structure for the addiction brain network [4]. It is well known that the cerebellum has a very high density of Cannabinoid receptor type 1 (CB1) receptors; however, little is known about its functional deficits due to chronic cannabis use. Motor adaptation is a cerebellum-dependent motor function that can be assessed with a visuomotor rotation (VMR) task and it might be altered in chronic cannabis users. Until now, there are no diagnostic tools that allow us to rate possible cerebellar impairments. We suggest that the VMR task could become a tool to assess cerebellar alterations due to chronic cannabis use (CCU). 

Given the current trend towards the tolerance/legalization of its medical and recreational uses [5,6], it is crucial to clarify how long-term cannabis consumption affects brain function and performance. Numerous studies have evaluated the behavioral effects of acute and non-acute cannabis use [7], showing effects of cannabis not only on cognitive processes such as attention and memory [8,9,10], but also on psychomotor tasks, where reaction times and the ability to inhibit motor actions are impaired [11,12]. In addition, acute cannabis consumption increases the risk of driving accidents two- to three-fold [13]. However, there is still no correlation between the known molecular effects of cannabis and changes in overt behavior. This hampers our ability to assess cannabis-induced levels of dysfunction and the associated risks of chronic use and cannabis addiction. 

Progress in understanding the consequences of cannabis requires linking its effects on the biological substrate with deficits in performance. THC [14] acts in the central nervous system through the CB1 receptor [15,16]. CB1 receptors are located in presynaptic terminals and have a regulatory effect on synaptic transmission [17,18]. The distribution and density of CB1 receptors in the brain may indicate which brain areas are more affected by the prolonged use of cannabis. Even though CB1 receptors are expressed all over the brain [19,20] (e.g., hippocampus, amygdala, striatum), their concentration is especially high in the molecular layer of the cerebellum [19]. In particular, cannabis consumption has been shown to down-regulate the CB1 receptors in the cerebellar molecular layer [21], affecting cerebellar plasticity mechanisms [22]. Indeed, chronic use of cannabis affects cerebellar-dependent behaviors both in animals and humans—specifically eye-blink conditioning, which is one of the basic paradigms for studying cerebellar associative learning [23]. CB1 knock-out mice and mice exposed to CB1 antagonists display a decreased acquisition of cerebellar-dependent eye-blink responses [24]. In humans, this result has been replicated in non-acute chronic cannabis users tested after 24 h of abstinence [25]. However, it is still not clear how deficits in eye-blink conditioning translate to easily administered diagnostic tests and instrumental activities of daily life. 

Motor adaptation is a cerebellar-dependent function that has not been studied in chronic cannabis users (CCUs). Through adaptation, the motor control system readjusts to changes in the dynamics of their musculoskeletal system and body configuration [26,27]. Motor adaptation occurs in automatically when a difference between the predicted trajectory and the observed movement exists. Motor adaptation is frequently studied with visuomotor rotation paradigms [28], where a specific perturbation creates a mismatch between the motor action and the observed outcome. Healthy individuals will gradually compensate for this mismatch and adjust to sizeable perturbations, without awareness of the process (implicit motor learning). One way to speed up adaptation is by introducing a strategy that enables participants to explicitly adjust their aiming direction to hit a given target, as Mazzoni and Krakauer did (MK task) [29]. By inserting a rule, healthy participants manage to immediately cancel aiming errors (explicit motor learning) but at the expense of setting the predicted trajectory and the actual trajectory in conflict. In other words, with practice, participants make increasingly larger errors (implicit motor learning occurs). In general, cerebellar pathologies induce reduced adaptation [30,31]. Specifically, in the MK task, cerebellar-ataxic patients, despite using an explicit strategy [32], performed “better” (with less systematic error), as implicit adaptation is impaired.

Given the above observations, we sought to assess the impact of chronic cannabis use (CCU) in motor adaptation using a VMR paradigm. We hypothesize that THC, acting as an exogenous agonist of the cannabinoid receptors, will diminish cerebellar plasticity. For this reason, we look for a measurable impact on motor adaptation—a cerebellar-dependent process that facilitates maintaining accurate control of motor behavior [27]—that can be studied with the VMR task [29,32]. We expect that, alike to cerebellar ataxic patients, chronic cannabis consumers will have reduced implicit motor adaptation and thus will perform more accurately than healthy controls on the VMR task. 

## 2. Experimental Section

### 2.1. Design

We conducted a case-control study. The study protocol is registered in clinicaltrials.gov, ID: NCT02816034. The study was approved by the Ethics Committee of Hospital Clínic de Barcelona (decision number HCB/2016/0018).

### 2.2. Participants

Study participants were between 18 and 50 years old. We included in the experimental group individuals following Diagnostic and Statistical Manual of Mental Disorders (DSM–V) criteria for Cannabis Use Disorder that screened positive for cannabis in the urine analysis performed the same day of their assessment. For the experimental and control groups, we recruited right-handed participants, without another active substance-use disorder (except of tobacco use disorder), with normal or corrected-to-normal vision. Participants with a cognitive impairment, such as mental retardation, or with psychotic disorders were excluded. Cannabis users were recruited among the patients of the Addiction Unit of a tertiary Hospital and their acquaintances. The sample included 17 CCUs and 18 healthy age-matched controls. All the participants signed an informed consent form before the initiation of the experiment. Participants were compensated for their effort with a voucher equivalent to a lunch ticket.

### 2.3. Procedures 

A clinical interview was performed by a psychiatrist and a psychologist with mental health and addiction expertise. Socio-demographic data, including gender and age, were collectedduring the clinical interviews. DSM-V criteria were used to diagnose patients with Cannabis Use Disorder. Furthermore, we gathered information about the frequency and quantity of cannabis consumed during the last week and the last 6 months using an ad hoc questionnaire. We used the first question of the Cannabis Use Disorder Identification Test (CUDIT) [33] to determine the frequency of cannabis use. In order to quantify cannabis use we used the Standard Joint Unit (1 SJU = 1 joint = 7 mg THC) [34]. The Scale for the Assessment and Rating of Ataxia (SARA) [35] was administered to assess ataxia. The SARA is a semi-quantitative scale, which has 8 items and assesses motor changes in gait, stance, sitting, speech disturbances, finger chase, nose-finger chase, fast alternating hand movements and heel–shin slide.

**Figure 1:**
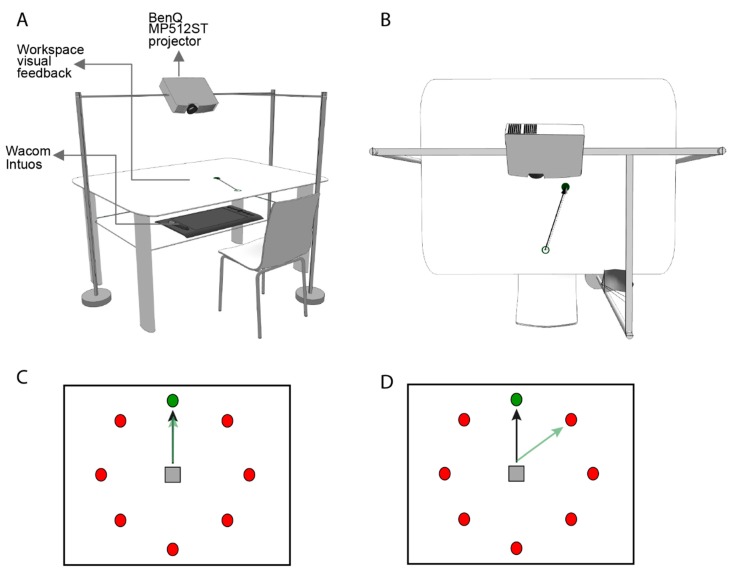
Perspective (**A**) and top view (**B**) of the experimental setup. Subjects were seated approximately 25 cm in front of a table with both hands occluded to vision. Movement trajectories were recorded through a digitizing tablet (Intuos Pro, Wacom, Saitama) located at waist height at the lower part of the table. We used a projector (BenQ MP512ST, Texas Instruments, Taoyuan, Taiwan), mounted 55 cm above the table, to display the behavioral tasks. The size of the projection was 46 ϗ 61 cm; data were sampled and stimuli displayed at 100 Hz. Prior to the experiment, we adjusted the seat in order to ensure that the distance between the center of the projection and the eyes of each participant was approximately 40 cm. (**C**) Visuomotor task. A centered home position (gray square) and eight circles (red) are projected over the surface, arranged in a circle. For each trial, a target appeared inside one of these eight circles (green circle). (**D**) During the Rotation and Rotation Strategy phases, the visual feedback (black arrow) of actual center–out reaching movements (green arrow) deviate by 45 degrees in a counter-clockwise direction.

Participants sat in front of a desk (Figure 1A,B) and were asked to control a cursor that was displayed on a screen, using an optical pen. Its position was recorded using a high-resolution digitizing tablet (Intuos3, Wacom, Saitama, Japan) at a sampling rate of 100 Hz. During the task, every trial required moving the stylus with the right hand over the digitization tablet from an initial central position. Visual feedback was projected on a surface placed in front of the subject. This surface occluded the subject’s view of the movements of their arm. The projection displayed eight circles over the surface, arranged in a circle (Figure 1C,D). For each trial, a target marked with a bulls-eye appeared inside one of these eight circles. Participants were instructed to perform a center–out reaching movement from the initial position towards the target with the pen. Participants moved as fast as possible to reach the target, which in turn turned red Then they then returned the pen to the start position. Depending on the phase of the experiment, the correct target was either the bulls-eye or the clockwise adjacent marker. 

The experimental protocol constisted of 7 phases and a total amount of 242 movements. These movements were grouped in 30 blocks of 8 trials and 1 of only 2trials. We replicated the protocol performed in cerebellar ataxic patients [32]. The protocol started with two baseline adaptation phases (B1 and B2) separated by a cognitive control phase (practice strategy, PS). Each baseline phase consisted of 24 movements (three blocks) where the participants moved towards the indicated target, and the visually projected cursor reproduced the participants’ movement trajectory. In the PS phase, participants were asked to aim to the marker adjacent to the target in the clock-wise direction (that is, the marker appearing at 45 degrees to the clock-wise direction). After these three phases, a visual rotation was introduced without warning during two trials. In these trials, the mapping of the movement-to-visual coordinates was altered such that the position of the displayed cursor was rotated 45 degrees counter-clockwise relative to the starting position. In order to force fast reaching movements, trials were repeated when movements were not completed within a time limit of 1.5 seconds after target appearance.

After these two trials, participants started the main experimental phase, i.e., the rotation plus strategy (RS) phase, where they were instructed to overcome the rotation by applying the strategy learned in the PS phase. The RS phase lasted 80 trials (10 blocks), that is, ten movements towards each target in random order. The last phase of the experiment started with a no-feedback washout (NF) phase, where participants were instructed not to apply the rotation strategy anymore, while they performed a series of 8 reaching movements, in the absence of visual feedback. Finally, in an 80 trials long washout phase (W), visual feedback was reinstalled in the absence of any rotation. The whole task lasted approximately 20 min.

### 2.4. Analysis

We measured performance in terms of the angular error—the angular difference between the executed movement and target directionsThis measure can be very noisy on a trial by trial basis, mostly because participants sometimes lapse and they apply the aiming rule incorrectly. For this reason we used the median angular error per block of 8 trials, noting that within a block all eight target positions were presented once. 

One patient and one control were removed from the final sample because of laterality problems and were not included in the analyses. An additional patient was discarded because her behavior was a clear outlier (3 SD) from the mean performance score and was not included in the analyses. Additionally, to avoid acute cannabis use effects on performance, we removed from the analysis all participants that had consumed cannabis within the 6 hours prior to performing the task (*n* = 4), as this interval has been used as a criterion for cannabis’ acute effects [36].

If not stated otherwise, sample estimates are reported as mean +/− standard deviation, and qualitative data are described as percentages. Chi-square test and t-tests were done to compare groups depending on the type of data. Bonferroni correction was performed for multiple comparisons and Cohen coefficient for effect size. 

## 3. Results

CCUs (*n* = 11, 4 women) had a mean age of 28.7 +/− 8.6 years old, which was age-matched by the control group (CG) (*n* = 17, 8 women; 30.7 +/− 7.1 years old). CCUs were diagnosed with a severe cannabis use disorder (≥ 6 criteria) and 75% were marijuana smokers. One subject was diagnosed with an attention deficit hyperactivity disorder in childhood but did not fulfill DSM-V criteria at the time of inclusion in the study. Experimental subjects showed significant differences in educational level compared to controls. For more details on clinical and sociodemographic characteristics, see Table 1.

**Figure 2:**
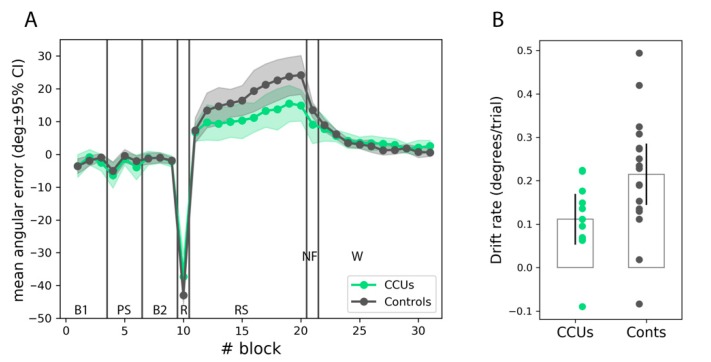
Motor adaptation in chronic cannabis users (CCUs) and control participants (Conts). (**A**): Evolution of the average error. Vertical lines separate different phases of the experiment, indicated by the labels: B1, first baseline period; PS: practice strategy; B2: second baseline period; R: rotation; RS: rotation plus strategy; NF: no feedback washout; W: regular washout. (**B**): Rate of error increase in the RS phase. Shaded areas and error bars indicate the 95% confidence interval of the mean.

The evolution of the angular error during the VMR task was our main behavioral outcome (Figure 2A). Cannabis users as well as controls demonstrated an average evolution of the angular error consistent with the expected interference between the use of an explicit strategy and an implicit adaptation process, as reported for healthy subjects in relevant studies [28,37]. Both groups experienced an increase in error (a drift towards positive angular errors) as trials accumulated in the RS phase. To assess implicit motor adaptation, we used both the maximum aiming error (degrees in cursor space) during the RS phase, or the *peak drift* [31], together with the *drift rate*, measured as the slope of the adaptation curve in the RS phase (in degrees per trial). To reduce the noise that was introduced by outlying trials, both measures were computed based on block scores, obtained as the median error for the eight trials in each block. Drift rate was significantly smaller for CCUs compared to controls (0.1 +/− 0.1°/trial vs. 0.2 +/− 0.1°/trial; t(26) = −2.2, *p* = 0.037, Cohen’s *d* = −0.9, 95% CI = (−1.8, −0.1)) and so was peak drift (19.3 +/− 6.7° vs. 27.4 +/− 11.6°; t(26) = −2.1, *p* = 0.048, Cohen’s *d* = −0.8, 95% CI = −1.7, −0.1), confirming the experimental hypothesis that chronic cannabis consumption can reduce motor adaptation. However, even though CCUs tended to display larger after-effects than controls, which were measured as the remnant error during the no-feedback washout phase (9.1 +/− 8.7° vs. 13.5 +/− 4.5°; t(26) = −1.8; *p* = 0.09), the difference did not reach significance. Notably, adaptation occurred very rapidly at the onset of the RS phase in comparison to the literature [38], which suggests that the use of continuous feedback might have provoked a fast explicit adaptation process in addition to implicit adaptation.

The difference in performance was not due to an intrinsic difference between CCUs and controls in unperturbed aiming, as both groups scored similarly in both baseline periods (−2.3 +/− 3.4° vs. −2.1 +/− 2.5°; t(26) = −0.1, *p* = 0.89 and −1.4 +/− 1.8° vs. −1.4 +/− 2.1°; t(26) = −0.1, *p* = 0.95 in B1 and B2, respectively). Differences were also minor in execution at either the onset or the offset of the PS phase (block 4: −6.4 +/− 5.8° vs. −5.0 +/− 5.2°; t(26) = −0.7, *p* = 0.51 and block 6: −4.0 +/− 5.7° vs. −2.1 +/− 3.1°; t(26) = −1.1, *p* = 0.26). No significant differences were found between CCUs and the CG in VMR task performance in terms of timings. 

Finally, even though the experimental and control groups did not differ significantly in terms of alcohol and tobacco consumption, one could not a-priori discard their involvement in the reduced adaptation observed in this study, as they both are psycho-active substances. To clarify this, we performed an ordinary least-squares analysis, where we added the levels of tobacco and alcohol consumption as possible confounds in addition to the group variable. Given the reduced sample size, the contrast between CCUs vs. controls only at the trend level (*p* = 0.10), but this was in striking contrast with the almost null influence observed both for tobacco (*p* = 0.75) and alcohol (*p* = 0.99). 

## 4. Discussion

This is the first study that analyzes the effects of chronic cannabis consumption on a visuomotor rotation task to assess the processes involved in motor adaptation. CCUs displayed impaired adaptation, manifested as lower aiming errors in the VMR task than controls.

Our results are consistent with our hypothesis that chronic cannabis use has an impact on cerebellar-dependent functions and, more specifically, on motor adaptation. The visuomotor adaptation paradigm has been used as a marker of cerebellar damage in ataxic patients, who show impaired implicit learning, putatively, due to disrupted forward internal models [32]. More specifically, cerebellar ataxic patients accumulate fewer aiming errors during the rotation and strategy (RS) phase, indicating a decreased motor adaptation [32]. It is interesting to note that the effect size of the original study with ataxic patients is very sizeable (Cohen’s d = −2.627), while the effect size on CCUs is moderate (Cohen’s d= −0.8). This is not surprising, as the clinical manifestation of cerebellar dysfunction of ataxia is severe, while cannabis-induced cerebellar alterations do not lead to a clinical phenotype (all the participants assessed scored zero on the SARA scale). However, we suggest that our results are clinically meaningful for two reasons: First of all, in several brain disorders, early alterations in the patient’s brain are present even decades before the first clinical symptoms appear [39,40]. Our paper shows an altered performance of CCUs in a visuomotor adaptation task, indicating subtle cerebellar alterations in the users´ brains. These results were obtained after excluding experimental participants under the acute effects of cannabis (on the suggestion of an anonymous reviewer). Interestingly, even though this reduced the sample size (from *n* = 33 to *n* = 28), statistical significance, and therefore the underlying effect size, increased (from d = −0.7 to −0.8). Our finding suggests that CCUs adapt less when they are not under the acute effects of cannabis. This deficit might be explained by the hypothesis of CB1 receptor down-regulation due to cannabis use [21]. More specifically, it has been shown that repeated cannabis exposure causes endocytosis of the endocannabinoid receptors, resulting in reduced activity of those receptors in the absence of their exogenous agonist. If this hypothesis explains our results, it would be plausible that acute cannabis use in CCUs may indeed hide an underlying impairment in cerebellar-dependent motor adaptation. Although our results show very subtle alterations compared to ataxic patients, these might be the first signs of a future clinically significant impairment that will only manifest with continuous heavy use [41]. Future longitudinal studies will clarify whether these early cerebellar alterations prelude long-term clinical symptoms. Secondly, it is noteworthy that even though, CCUs show no measurable motor deficits clinically, they share a certain degree of alterations in motor adaptation with cerebellar ataxic patients. Future research will show the reliability of the task as a potential tool for assessing brain alterations, as well as whether the latter are present exclusively in cannabis users or they are part of addiction-induced brain alterations. 

Globally, these results indicate that VMR tasks might be a simple, non-invasive and novel way to start exploring a basic cerebellar function in CCUs. The cerebellum is crucial for motor adaptation, among other functions. As implicit learning is an essentialskill for everyday functioning, our findings are the first step towards understanding more complex cerebellar deficits. Our results are in line with the increasingly recognized role of the cerebellum in higher cognitive functions and emotional processes, and a recent review that points out a cerebellar involvement in cannabis addiction [42]. Future research should examine whether there are changes in the performance of users after cessation of use, as well as if this task can capture cerebellar alterations in individuals dependent on other substances, such as tobacco, alcohol, amphetamines, etc. 

This study has limitations that have to be taken into consideration when interpreting the results. First of all, the small sample size might have compromised the power of the study, so further studies should increase the sample size and also explicitly indicate in the protocol that participants are required to refrain from cannabis use for at least 6 hours prior to the study. Regardless of the small sample size, the moderate effect size might be due to the fact that CCUs do not show any clinically significant motor deficits (SARA score equals to 0), unlike ataxic patients. Secondly, significant differences were found between groups in terms of educational level. While we propose that educational level should not be an important confounder, due to the implicit nature of the task, tobacco and alcohol use might have had an effect on performance. Indeed, even though an ordinary least-squares analysis showed inexistent correlation with performance in the present study, one should take into account for future studies that reduced adaptationmight arise due to the synergistic effects of all substances, and not exclusively of cannabis. Thirdly, even if the small differences that we found in motor adaptation are representative of cerebellar alterations in CCUs, no causation can be inferred, as the brain differences might have been preceded cannabis use. Future longitudinal studies should clarify this matter. Finally, in previous studies where participants only received end-point position feedback, the systematic aiming error accumulated slowly during the 80 trials of the RS phase. However, we opted for providing participants with continuous feedback, evaluating the angular orientation using the initial part of the trajectory. The increased level of feedback has introduced discrepancies with the literature. First, as intended, it may have accelerated (implicit) adaptation, consistently with Taylor et al. [38], where a different VMR task was used. Indeed, the drift rate in our control group (0.2°/trial) is twice as fast as in Taylor et al. [32] (circa 0.1°/trial). Secondly, it has produced a shift in the maximum peak drift, which here reached 27.2° for the control group whereas in previous experiments it remained near 10° [29,32]. This increased peak drift might be due to fast explicit processes [38], as suggested by the high level of adaptation already manifested in the first block of the RS phase. However, we expect to have minimized the influence of the explicit learning in our main result by reporting the drift rate, which was computed taking into account the median performance for each one of the ten blocks of eight trials of the RS phase. In other words, our measure of drift rate is not affected by the high level of adaptation which had already been reached at the first block of the RS phase. Besides, even though we report a tendency in the after-effects that is consistent with our hypothesis of decreased implicit adaptation in CCUs, the difference only reached trend-level significance (*p* < 0.09). Hence, the replication of these results with a bigger sample size may be needed to confirm that the difference in performance reported here is due to putatively-cerebellar implicit motor learning. Indeed, a possibility to avoid involving explicit processes would be using newer VMR paradigms more precisely targeted at measuring implicit learning [43]. 

## 5. Conclusions

Taken altogether, CCUs showed a moderately decreased motor adaptation compared to controls, assessed with the VMR task. This tool is easy to implement and might be able to detect subtle changes in brain function before clinical symptoms of damage occur. Future research will clarify whether the VMR task can emerge as a potential assessment tool of brain dysfunction secondary to chronic cannabis consumption.

## Figures and Tables

**Table 1:** Sociodemographic and clinical characteristics of the experimental (chronic cannabis users) and control groups.

	Experimental (*n* = 11)				Control (*n* = 17)					
	*n*	%	Mean	SD	*n*	%	Mean	SD	χ^2^/t/fisher	*p*-Value
Men	7	63.6			9	52.9			0.3	0.6
Age			28.3	8.1			30.7	7.1	−0.8	0.41
Academic Level									13.7	<0.001
High school	8	72.7			1	5.9				
University	3	27.3			16	94.1				
Civil Status									0.05	0.8
Single	8	72.7			13	76.5				
Married	3	27.3			4	23.5				
Age at first cannabis consumption			16.6	3			15	2		
Age at regular cannabis use			19	2.1						
Regular cigarette smokers (yes)	4	36.4			1	6.3			0.134	0.07
Frequency of Cannabis use last 6 months										
4 to 6 times per week	2	18.2								
Daily	9	75.0								
SJU/day last 6 months			3.7	3.7						
Frequency of Cannabis use last week										
Never	2	18.2								
2 to 3 times per week	1	69.1								
4 to 6 times per week	2	18.2								
Daily	6	54.5								
SJU/day last week			2	2.2						
Standard drink units per week			6.7	8.3			2.9	3.9	1.6	0.1
SARA			0				0			

SD: Standard Deviation; SJU: Standard Joint Unit (1 SJU = 7 mg THC); SDU: Standard Drink Unit (1 SDU = 10 gr of pure alcohol). SARA: Scale for the Assessment and Rating of Ataxia. Bonferroni correction = *p*-value = 0.006.
